# Population structure of ocular *Streptococcus pneumoniae* is highly diverse and formed by lineages that escape current vaccines

**DOI:** 10.1099/mgen.0.000763

**Published:** 2022-03-07

**Authors:** Camille Andre, John Rouhana, Suelen Scarpa de Mello, Gabriela Rosa da Cunha, Andrew G. Van Camp, Michael S. Gilmore, Paulo J.M. Bispo

**Affiliations:** ^1^​ Infectious Disease Institute, Boston, MA 02114, USA; ^2^​ Department of Ophthalmology, Massachusetts Eye and Ear, Harvard Medical School, Boston, MA 02114, USA; ^3^​ Department of Microbiology and Immunobiology, Harvard Medical School, Boston, MA, 02115, USA; ^†^​ Present address: Clinical Laboratory, Hospital Ernesto Dornelles, Porto Alegre, Brazil

**Keywords:** *S. pneumoniae*, ocular infections, genomic epidemiology, polysaccharide capsule, pneumococcal conjugative vaccine (PCV)

## Abstract

*
Streptococcus pneumoniae* is a leading cause of ocular infections including serious and sight-threatening conditions. The use of pneumococcal conjugate vaccines (PCV) has substantially reduced the incidence of pneumonia and invasive pneumococcal diseases, but has had limited impact on ocular infections. Additionally, widespread vaccine use has resulted in ongoing selective pressure and serotype replacement in carriage and disease. To gain insight into the population structure of pneumococcal isolates causing ocular infections in a post-PCV-13 time period, we investigated the genomic epidemiology of ocular *S. pneumoniae* isolates (*n*=45) collected at Massachusetts Eye and Ear between 2014 and 2017. By performing a series of molecular typing methods from draft genomes, we found that the population structure of ocular *S. pneumoniae* is highly diverse with 27 sequence types (grouped into 18 clonal complexes) and 17 serotypes being identified. Distribution of these lineages diverged according to the site of isolation, with conjunctivitis being commonly caused by isolates grouped in the Epidemic Conjunctivitis Cluster-ECC (60 %), and ST448 (53.3 %) being most frequently identified. Conversely, *S. pneumoniae* keratitis cases were caused by a highly diverse population of isolates grouping within 15 different clonal complexes. Serotyping inference demonstrated that 95.5 % of the isolates were non-PCV-13 vaccine types. Most of the conjunctivitis isolates (80 %) were unencapsulated, with the remaining belonging to serotypes 15B, 3 and 23B. On the other hand, *S. pneumoniae* causing keratitis were predominantly encapsulated (95.2 %) with 13 different serotypes identified, mostly being non-vaccine types. Carriage of macrolide resistance genes was common in our ocular *S. pneumoniae* population (42.2 %), and usually associated with the *mefA +msrD* genotype (*n*=15). These genes were located in the Macrolide Efflux Genetic Assembly cassette and were associated with low-level *in vitro* resistance to 14- and 15-membered macrolides. Less frequently, macrolide-resistant isolates carried an *ermB* gene (*n*=4), which was co-located with the *tet*M gene in a Tn-916-like transposon. Our study demonstrates that the population structure of ocular *S. pneumoniae* is highly diverse, mainly composed by isolates that escape the PCV-13 vaccine, with patterns of tissue/niche segregation, adaptation and specialization. These findings suggest that the population structure of ocular pneumococcus may be shaped by multiple factors including PCV-13 selective pressure, microbial-related and niche-specific host-associated features.

## Abbreviations

CC, clonal complexes; ECC, epidemic conjunctivitis cluster; IPD, invasive pneumococcal disease; MEE, Massachusetts Eye and Ear; MIC, minimum inhibitory concentration; MLST, multilocus sequence typing; PCV, pneumococcal conjugative vaccine; ST, sequence type.

## Impact Statement

*Streptococcus pneumoniae* is the leading cause of community-acquired pneumonia, bacteremia and meningitis, as well as non-invasive infections including serious and sight-threatening ocular infectious diseases. Massive administration of vaccines targeting the polysaccharide capsule has substantially reduced morbidity and mortality associated with pneumococcal diseases, but has had limited impact on ocular infection incidence. By performing a series of molecular typing methods from draft genomes we found that the population structure of ocular *S. pneumoniae* is highly diverse and mainly composed by isolates that scape the 13-valent pneumococcal conjugate vaccine. We also found differences in the microscale biogeography of ocular pneumococcal infections, with conjunctivitis being commonly caused by unencapsulated isolates mainly associated with a distinct cluster of closely related lineages that seem to be confined to the conjunctiva. Conversely, pneumococcal keratitis was caused by a highly diverse population of non-vaccine encapsulated isolates grouping within 15 different clonal complexes that are scattered across the phylogenomic tree, and interspersed with strains originating from other body sites. This work provides new insights into the population structure of ocular *S. pneumoniae* that will serve as a foundation for performing large-scale studies to better understand the epidemiology of ocular pneumococcal infections, and inform the design of next-generation vaccines to prevent, and help decrease the economical and health burden of ocular pneumococcal infections.

## Data Summary

This Whole Genome Shotgun project has been deposited at DDBJ/ENA/GenBank under the BioProject number PRJNA715222.

## Introduction

*Streptococcus pneumoniae* is an inhabitant of the upper respiratory tract and a leading cause of invasive infections including pneumonia, bacteremia and meningitis [1]. This organism is also a common cause of non-invasive infections such as otitis [2] and ocular infections, including conjunctivitis [3], keratitis [4] and endophthalmitis [5]. In the USA, *S. pneumoniae* ranks among the top five most common causes of ocular surface infections, being associated with around 6–7.6 % of bacterial conjunctivitis cases in all age groups [6–8], nearly 30 % in children [9] and 6–10 % of keratitis cases [8, 10]. The burden of pneumococcal ocular infections seems to be even higher in the developing world, especially southern Asia, where *S. pneumoniae* is the leading cause of bacterial keratitis being associated with up to 36 % of all cases [11].

Production of a polysaccharide capsule enables *S. pneumoniae* to evade phagocytosis by inhibiting complement-mediated opsonization and is a key virulence factor in invasive pneumococcal disease (IPD) [12]. Currently, more than 90 capsule serotypes have been identified [13], some of which are targeted by the pneumococcal conjugate vaccines (PCV). PCV-13, which protects against 13 different serotypes (1, 3, 4, 5, 6A, 6B, 7F, 9V, 14, 19A, 19F, 18C and 23F) has been recommended as part of the childhood immunization programme since 2010 in the USA [14]. PCV-20, which offers protection against seven additional serotypes (8, 10A, 11A, 12F, 15B, 22F and 33F), has been recently approved by the US Food and Drug Administration for use in adults only. Widespread vaccination has significantly reduced morbidity and mortality in IPD [14, 15], but seems to have a smaller impact in preventing pneumococcal ocular diseases since the common lineages colonizing the ocular surface or causing conjunctivitis are often not covered by the current vaccines [6, 16–18].

In addition to being a common cause of sporadic bacterial conjunctivitis in all age groups, a costly disease that results in high economic burden [19] *S. pneumoniae* has been also associated with several outbreaks of this disease in the USA [3, 20, 21]. We previously reported that *S. pneumoniae* conjunctivitis is mainly caused by a highly divergent group of unencapsulated strains termed Epidemic Conjunctivitis Cluster (ECC), which are not targeted by current vaccine strategies [22]. ECC strains carry a variety of gene clusters that are absent or substantially different in encapsulated strains from invasive infections, and are thought to play an important role in the pathogenesis of epidemic conjunctivitis. This bifurcation in phylogeny and hyper-niche specialization of ECC is thought to have been driven by selective forces present in the unique environment of the ocular surface wet mucosa, which may play a role in community assemblage and dynamics impacting the microscale biogeography of conjunctivitis [23].

Because of the ongoing vaccine selective pressure, and because in addition to conjunctivitis, *S. pneumoniae* is a leading cause of infectious keratitis [11], we sought to determine whether ECC continue to predominate in conjunctivitis, and whether their tropism extends to other ocular tissues. By creating a phylogeny, and correlating that with a series of molecular typing methods using data extracted from draft genome sequences, we found that the population structure of ocular *S. pneumoniae* diverges according to the site of isolation, with ECC-related isolates being confined to the conjunctiva. Keratitis cases examined here are caused by a highly diverse population of mainly encapsulated *S. pneumoniae* lineages that escape the currently available PCV vaccines.

## Methods

### Bacterial isolates

From 2014 to 2017, 45 consecutive and non-duplicate *S. pneumoniae* were isolated from multiple ocular sites of infections (keratitis *n*=21, conjunctivitis *n*=15, endophthalmitis *n*=4, dacryocystitis *n*=4 and orbital cellulitis *n*=1) and included in this work. Protocols for obtaining discarded isolates with waived informed consent were approved by the MEE Institutional Review Board. Primary clinical specimens collected from multiple ocular infection sites were obtained by the attending ophthalmologist or resident following institutional guidelines and submitted to the clinical microbiology laboratory for processing. Suspected *S. pneumoniae* colonies were identified using the MicroScan WalkAway system (Beckman Coulter, Brea, CA) following the manufacture’s protocol. Isolates were stored at −80 °C in Microbank cryopreservative tubes (ProLab Diagnostics). Frozen isolates were cultured on 5 % sheep blood agar plates (BD Biosciences, San Jose, CA) and incubated at 37 °C with 5 % CO_2_.

### Genome sequencing and assemblies

To understand the population structure of *S. pneumoniae* causing ocular infections, isolates included in this study were submitted to whole-genome sequencing. Total DNA was purified from an overnight pure culture in 5 ml Todd Hewitt broth using the DNeasy DNA extraction kit (Qiagen, Valencia, CA). DNA quality was verified on a Bio-Tek Synergy two microplate reader (Winooski, VT) prior to quantification using a Qubit fluorometer and dsDNA High-Sensitivity assay kit (Invitrogen, Carlsbad, CA). Library preparation for Illumina sequencing was carried out using the Nextera XT DNA Library Preparation kit (Illumina, San Diego, CA), according to the manufacturer’s specifications. Quality and quantity of each sample library was measured on a TapeStation instrument (Agilent Technologies, Santa Clara, CA). The genomes were sequenced as 2×100 bp or 250 bp reads on an Illumina HiSeq sequencer, according to the manufacture’s specifications with a minimum depth of coverage of 30× (ranging from 30× to 855×; median 243×). Sequence reads were assembled *de novo* using CLC Genomics Workbench (CLC Bio, Cambridge, MA). Samples with sequence reads below a quality score of 25 at any position and assemblies that were not between 1.8 to 2.4 Mb were re-sequenced. This Whole Genome Shotgun project has been deposited at DDBJ/ENA/GenBank under the BioProject number PRJNA715222.

### Phylogenetic tree

To generate a phylogeny, the core genomes of 45 ocular samples and 29 references from Valentino *et al*. [22] were aligned using Parsnp from Harvest v1.1.2 using standard settings [24]. Recombination was removed from the alignment using the PhiPack function from Parsnp [25]. Before recombination was removed, the alignment was 2120235 bp long, and after recombination was removed, the alignment was 2106297 bp long. A maximum-likelihood tree was generated using IQ-TREE, which found 26494 parsimony-informative sites in the alignment, with 1000 bootstraps, *Streptococcus mitis* strain B6 as an outgroup, and random seed number 545212 [26–28]. The phylogeny was visualized and annotated with iTol v4 [29].

### Prediction of sequence types, antibiotic resistance genes and serotypes

We used the Center for Genomic Epidemiology pipeline to obtain confirmation of species identification, sequence type (ST) (as determined by the current multilocus sequence typing scheme for *S. pneumoniae *), and to identify the pool of acquired antibiotic resistance genes in each genome using the ResFinder algorithm [30]. A cutoff of 99 % identity at the nucleotide sequence level was used for detection of antibiotic resistance genes. Beta-lactam resistance was genotypically predicted by using a penicillin-binding protein (PBP) typing system [31], based on sequence signatures in the transpeptidase domains. Clonal complexes (CC) were determined using the MLST data by the goeBURST algorithm (http://www.phyloviz.net/goeburst/). Determination of newly proposed nomenclature by the GPS project of Global Pneumococcal Sequence Clusters (GPSCs) for internationnaly distributed pneumococcal lineages [32] was done using the PathogenWatch application (https://pathogen.watch/). Capsule serotype was predicted from genome sequences using PneumoCaT (Pneumococcal Capsular Typing) v1.2.1. The genetic Tn916-like and Megacassette environments were built by using Easyfig v2.2.5.

## Results

### The population structure of ocular *S. pneumoniae* diverges according to the site of infection

**Fig. 1.**
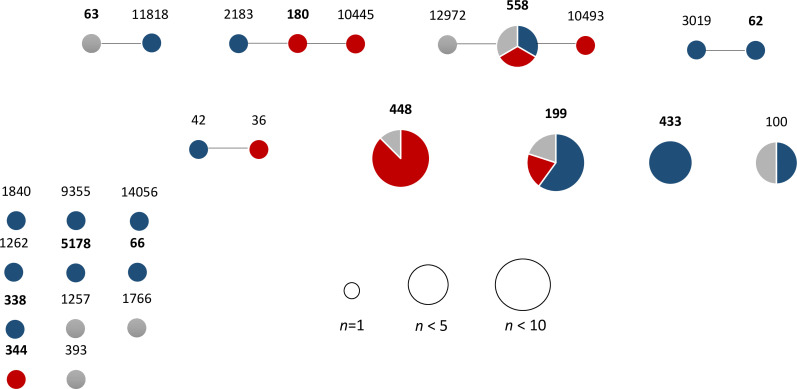
goeBURST population analysis of MLST allelic profiles of 45 *S *. *pneumoniae* isolates, organized by site of infection. Circle sizes are reflective of ST frequency, and are coloured based on the percentage of isolates collected from keratitis (blue), conjunctivitis (red) or others (grey). Bold numbers are indicative of the founder ST of a clonal complex.

**Fig. 2.**
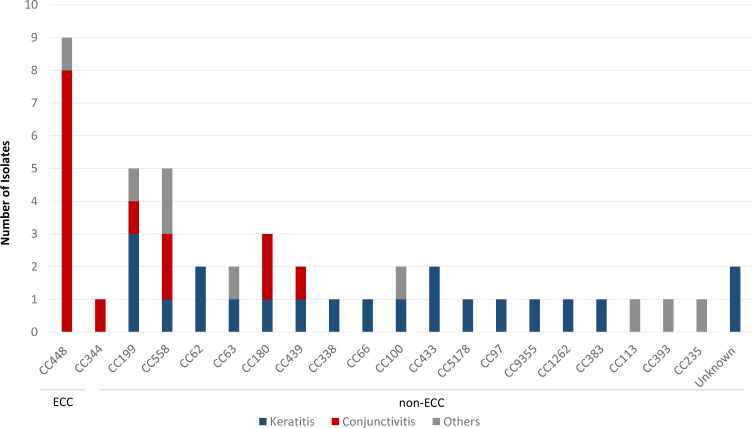
Distribution of clonal complexes among 45 *S *. *pneumoniae* ocular isolates according to the site of infection. ECC, Epidemic Conjunctivitis Cluster.

**Fig. 3.**
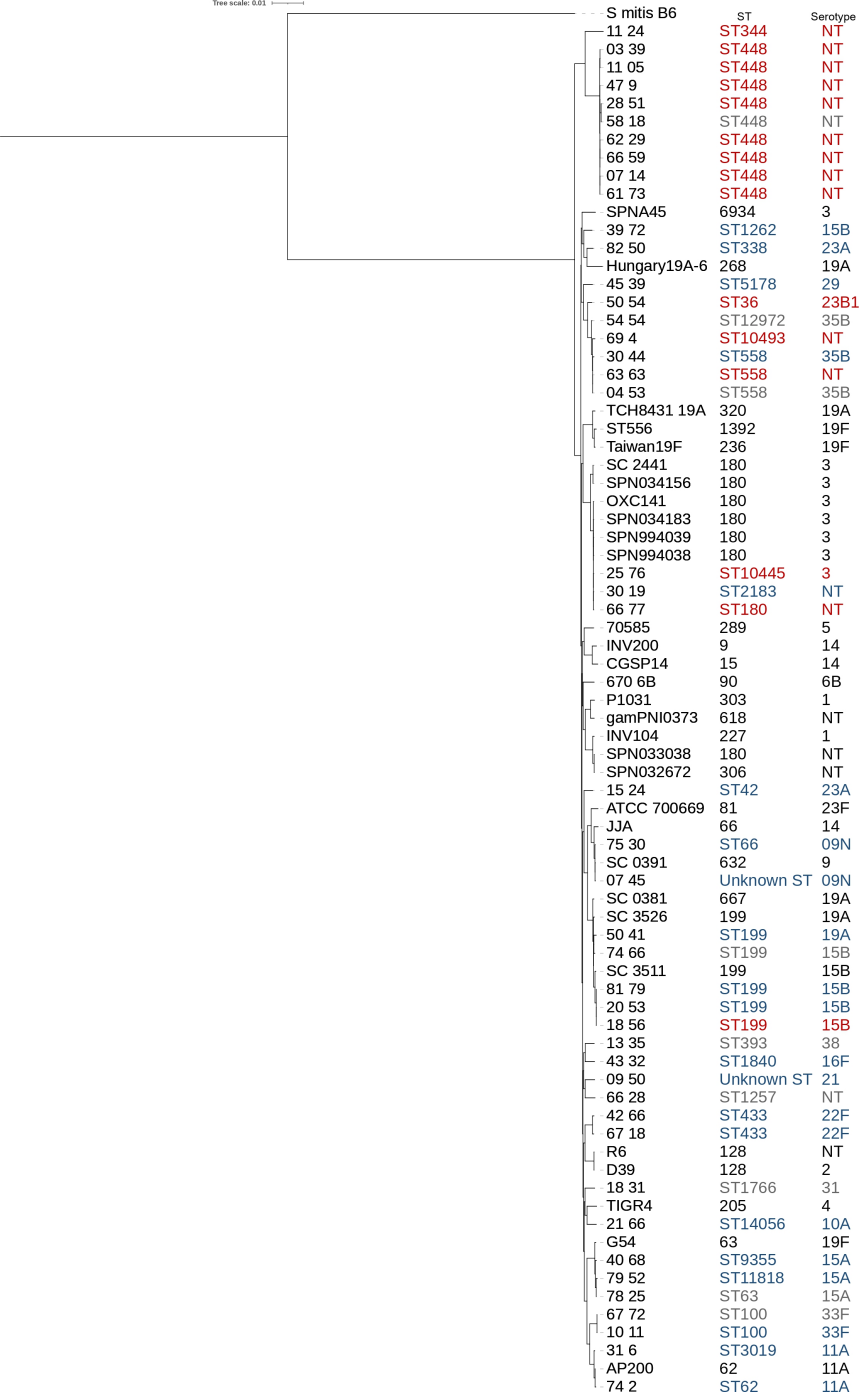
SNP-based maximum-likelihood phylogenetic tree of the *S. pneumoniae* ocular population generated from a core-genome alignment. Bootstrapping was performed with 1000 iterations. Isolates are coloured based on their source of isolation including keratitis (blue), conjunctivitis (red) and others (grey). All comparator genomes included in the tree were coloured black.

To investigate whether the pathogenesis of dominant ECC lineages extents to various ocular tissues/niches or if it is confined to the conjunctival mucosa, we generated draft genomes of 45 *S *. *pneumoniae* isolates from ocular infections, including keratitis (*n*=21, 46.7 %), conjunctivitis (*n*=15, 33.3 %), endophthalmitis (*n*=4, 8.9 %), dacryocystitis (*n*=4, 8.9 %) and orbital cellulitis (*n*=1, 2.2 %). Genome sequence-based multilocus sequence typing (MLST) analysis of isolates from all ocular sites identified 27 unique STs grouped into 20 clonal complexes (CCs), revealing a highly diverse population of isolates (Table 1, Fig. 1). The most common CCs were represented only by a handful of isolates and included CC448 (20 %, *n*=9), CC199 (11.1 %, *n*=5) and CC558 (11.1 %, *n*=5) (Table 1, Fig. 1). Moreover, the distribution of CCs diverges across different infection sites, with a pattern of lineage predominance in conjunctivitis. Isolates grouping within the ECC (*n*=10) appear highly adapted and confined to the conjunctival mucosa only. Non-typeable ECC isolates were the most common causes of conjunctivitis in our population (9/15; 60 %), with ST448 (8/15; 53.3 %) being most frequently identified (Table 1, Figs 1 and 2). These isolates were mainly isolated from conjunctivitis (*n*=9) and belonged to ST448 (*n*=9) and ST344 (*n*=1). One ST448 (isolate 58–28) was isolated from the conjunctival discharge of a patient presenting with scleral buckle extrusion and orbital cellulitis, and was not considered as conjunctivitis. A second ST448 isolate (isolate 7 14) was recovered from a corneal scraping of a patient that presented with an initial chief complaint of conjunctivitis that evolved with a small peripheral corneal infiltrate, and was considered a conjunctivitis case with a possible secondary marginal keratitis. Conversely, *S. pneumoniae* keratitis were caused by a highly diverse population of encapsulated isolates grouping within 15 different CCs that were somewhat evenly distributed across the cases (Table 1, Figs 1 and 2). The diversity of this population was also confirmed by assigning our isolates to the Global Pneumococcal Sequence Clusters (GPSCs). In total 17 unique GPSCs were found (compared to 27 by MLST), with GPSC 60 (representing ST448) being over represented in conjunctivitis, and the keratitis population being highly diverse and containing 13 unique GPSCs (Table S1, available in the online version of this article). To correlate the MLST-based population structure findings with the phylogenomics of this isolate collection, a SNP-based maximum-likelihood tree was generated based on a core-genome alignment for genes shared among all the 45 ocular *S. pneumoniae* genomes and 29 comparator strains isolated from other body sites (Fig. 3). Our ECC-related isolates (ST448 and ST344) formed a deeply resolved group, as we previously observed [22]. Oppositely, lineages causing pneumococcal keratitis did not cluster together, were scattered across the tree and interspersed with strains that cause infections in other body sites, confirming that they form a highly diverse population of unrelated isolates as determined by MLST. Importantly, the SNP-based phylogenetic tree was reconstructed after removal of recombinant DNA segments using the PhiPack function from Parsnp, demonstrating that this population structure was not driven by recombination.

**Table 1.** Clonal complex, sequence type and serotypes of the *S. pneumoniae* ocular isolates according to their isolation sites

Clonal complex (no. of isolates)	STs (no. of isolates)	Serotypes (no. of isolates)	no. of isolates from:		
			Keratitis (*n*=21)	Conjunctivitis (*n*=15)	Others (*n*=9)
**ECC isolates**					
CC448 (9)	ST448 (9)	NT (9)	–	8	1^a^
CC344 (1)	ST344 (1)	NT (1)	–	1	–
**Non-ECC isolates**					
CC199 (5)	ST199 (5)	15B (4)	2	1	1^b^
		19A (1)	1	–	–
					
CC558 (5)	ST558 (3)	35B (2)	1	–	1^c^
		NT (1)	–	1	–
	ST12972 (1)	35B (1)	–	–	1^b^
	ST10493 (1)	NT (1)	–	1	–
					
CC62 (2)	ST3019 (1)	11A (1)	1	–	–
	ST62 (1)	11A (1)	1	–	–
					
CC63 (2)	ST63 (1)	15A (1)	–	–	1^c^
	ST11818 (1)	15A (1)	1	–	–
					
CC180 (3)	ST2183 (1)	NT (1)	1	–	–
	ST10445 (1)	3 (1)	–	1	–
	ST180 (1)	NT (1)	–	1	–
					
CC66 (1)	ST66 (1)	09 N (1)	1	–	–
					
CC338 (1)	ST338 (1)	23A (1)	1	–	–
					
CC439 (2)	ST42 (1)	23A (1)	1	–	–
	ST36 (1)	23B (1)	–	1	–
					
CC100 (2)	ST100 (2)	33F (2)	1	–	1^c^
					
CC433 (2)	ST433 (2)	22F (2)	2	–	–
					
CC5178 (1)	ST5178 (1)	29 (1)	1	–	–
					
CC97 (1)	ST14056 (1)	10A (1)	1	–	–
					
CC9355 (1)	ST9355 (1)	15A (1)	1	–	–
					
CC1262 (1)	ST1262 (1)	15B (1)	1	–	–
					
CC383 (1)	ST1840 (1)	16F (1)	1	–	–
					
CC113 (1)	ST1766 (1)	31 (1)	–	–	1^b^
					
CC393 (1)	ST393 (1)	38 (1)	–	–	1^b^
					
CC235 (1)	ST1257 (1)	NT (1)	–	–	1^c^
					
Unknown CC (2)	Unknown ST (2)	09 N (1)	1	–	–
		21 (1)	1	–	–

Isolated from ^a^cellulitis, ^b^endophthalmitis, ^c^dacryocystitis.

### Current pneumococcal vaccines do not target ocular *S. pneumoniae*

**Fig. 4.**
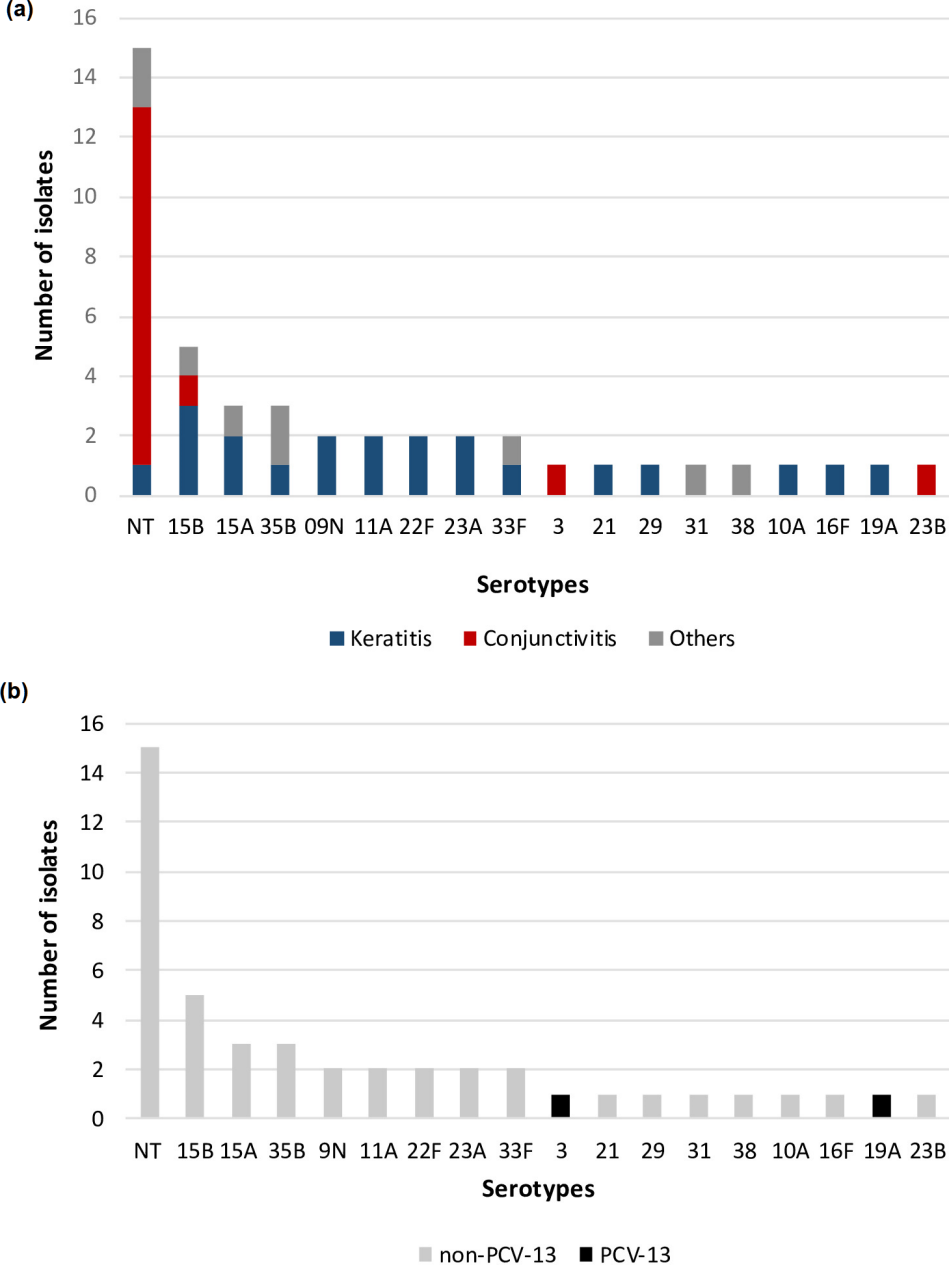
Distribution of serotypes according to (a) the site of infection, or (b) serotype inclusion in the Pneumococcal Conjugate Vaccine-13 (PCV-13). Grey bars represent serotypes not covered PCV-13, and black bars represent serotypes covered by PCV-13.

To analyse the distribution of ocular *S. pneumoniae* capsular serotypes according to the disease, and to determine the influence of the PCV-13 on this distribution, we extracted presumptive capsular serotyping data from draft genome sequences using PneumoCat. In agreement to our previous findings [22], most of the conjunctivitis *S. pneumoniae* isolates (12/15; 80%) were unencapsulated, with the remaining belonging to serotypes 15B, 3 and 23B (Fig. 4a). On the other hand, *S. pneumoniae* causing keratitis were predominantly encapsulated (20/21; 95.2%). The genotypically heterogeneous population of keratitis isolates, as determined by MLST analysis, also presents with a high degree of diversity at the capsule serotype level. Thirteen different capsule operon types were identified among these isolates and included the following inferred serotypes: 15B, 11A, 15A, 9 N, 23A, 22F, 35B, 33F, 29, 21, 19A, 16F and 10A (Table 1, Fig. 4a).

Although the introduction of PCVs in the US childhood immunization schedule has substantially decreased the rates of IPD and nasopharyngeal colonization by vaccine serotypes, this selective pressure is known to result in increased carriage by replacement non-vaccine serotypes [33]. In our sample, which represents a post-PCV-13 population, 95.5 % (43 out of 45) of the isolates were non-vaccine types (Fig. 4b). Conjunctivitis continues to be mainly caused by unencapsulated isolates that do not produce a polysaccharide capsule, a key virulence factor that is targeted by the PCV. Among the typeable conjunctivitis isolates, only one carried a capsule operon that was identified as the PCV-13 serotype 3. Pneumococcal keratitis, a sight-threatening disease that was mainly caused by encapsulated *S. pneumoniae* that theoretically could be prevented through vaccination, was primarily associated (95.2%, 20/21) with non-PCV-13 serotypes (Fig. 4a, b).

### Ocular *S. pneumoniae* frequently carries macrolide resistance genes

**Fig. 5.**
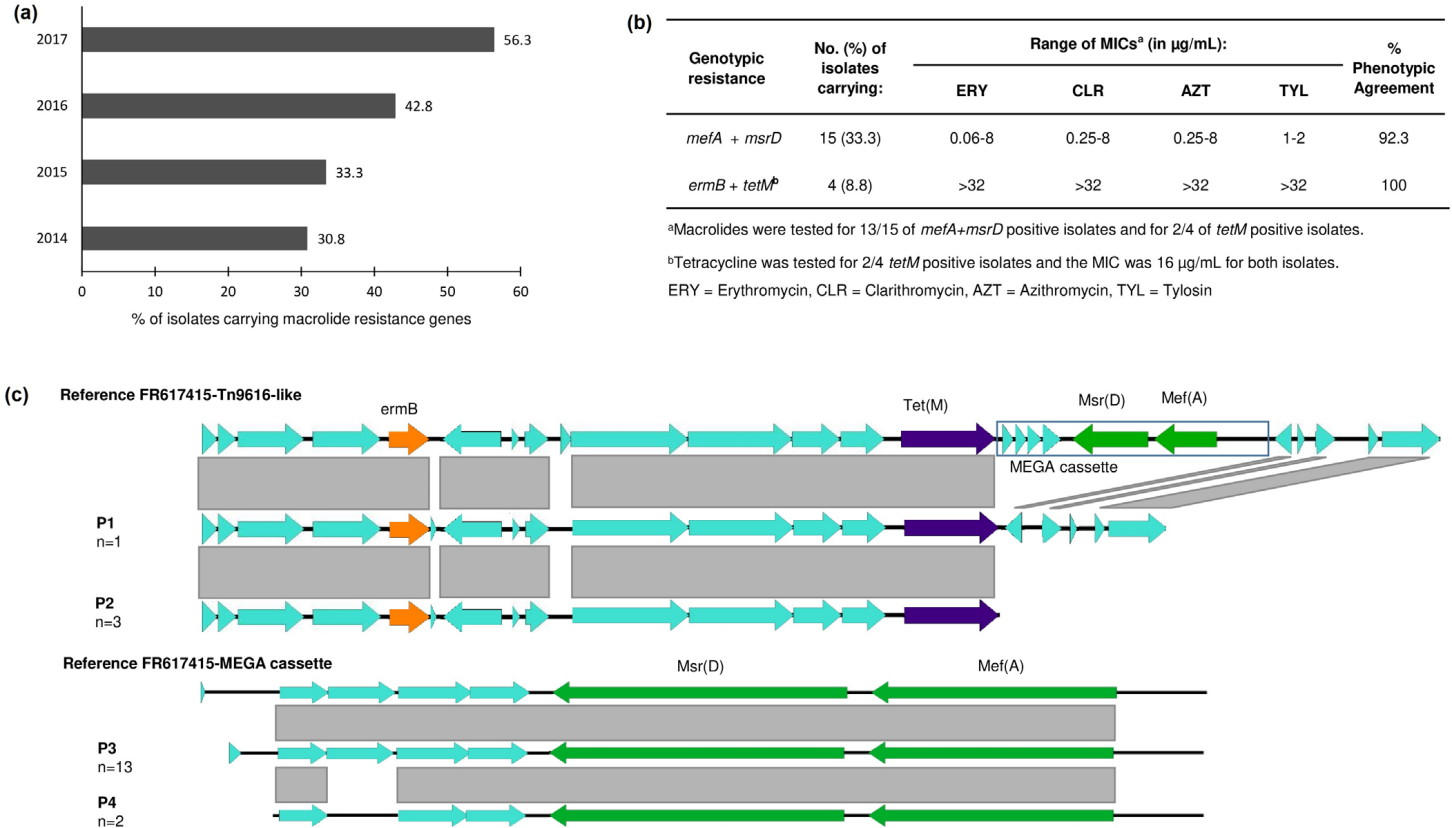
(a) Percentage of *S. pneumoniae* isolates carrying macrolide resistance genes from 2014 to 2017. (b) Distribution of macrolide resistance genotypes and phenotypes. (c) Synteny of macrolide resistance elements in ocular *S. pneumoniae* isolates as compared to a reference sequence (FR617415). For both, Tn916-like transposon and mega cassette, two different synteny patterns were found, named here as P1 and P2; and P3 and P4, respectively.

To characterize the genotypic antibiotic resistance profile of ocular *S. pneumoniae* isolates, we used their draft genomes to identify the pool of acquired resistance genes using ResFinder. Carriage of macrolide resistance genes was the most common (19/45; 42.2 %), and usually associated with the *mefA+msrD* (*n*=15) genotype (Fig. 5b). These genes are located in the Macrolide Efflux Genetic Assembly (mega) cassette (Fig. 5c) and encode an efflux transport system of the ATP-binding cassette superfamily that confers resistance to 14- and 15-membered macrolides [34]. In agreement with this, most of our isolates carrying the *mefA+msrD* genes (92.3 %) exhibited low-level *in vitro* resistance to erythromycin, azithromycin and clarithromycin (MIC ranging from 0.06 to 8 µg ml^−1^, Fig. 5b), and were susceptible to the 16-membered macrolide, tylosin. Less frequently, macrolide-resistant isolates carried *ermB* gene (*n*=4), encoding ribosomal methylase. *erm*B carriage resulted in high-level resistance to the macrolides tested (MIC>32 µg ml^−1^). The percentage of isolates carrying a macrolide resistance element increased over the period of our study from 30.8 % in 2014–33.3 % in 2015, 42.8 % in 2016 and 56.3 % in 2017 (Fig. 5a). A small number of isolates harboured the *tetM* gene (*n*=4), which confers resistance to tetracycline. In these isolates, the *ermB* gene was also present and they were localized to a *Tn916*-class transposon (Fig. 5c). No significant difference was found in the rate of carriage of acquired antibiotic resistance elements between encapsulated (43.3 %) and unencapsulated (46.7 %) isolates (data not shown). Genomic-based prediction of beta-lactam resistance demonstrated that most isolates (88 %) for which the model was able to generate results were sensitive to these antimicrobials, with the remaining predicted to be resistant to cefuroxime (some of these also with intermediate resistance to amoxicillin) (Table S1). Phenotypically, all isolates were susceptible to antibiotics commonly used for topical treatment of conjunctivitis and keratitis, including levofloxacin and vancomycin (data not shown).

## Discussion

Pneumococcal ocular infections occur in a variety of eye tissues and compartments, each with their specificities and unique mechanisms of protection [35–37]. These differences appear to shape the population structure of infection-derived ocular *S. pneumoniae *. Previously, we found that pneumococcal conjunctivitis in the USA is mainly caused by a highly specialized clade of unencapsulated strains termed Epidemic Conjunctivitis Cluster (ECC) [22]. Because the current vaccines target the polysaccharide capsule, these strains escape the selection elicited by immunization. Our results show that ECC tropism is specific to the conjunctiva subdomain of the ocular surface, with keratitis being caused by a highly diverse population of mainly encapsulated, non-ECC *S. pneumoniae* lineages, but lineages that are also not covered by PCV-13.

The molecular basis for this bifurcation of the population between two ocular surface tissues is not known. Our previous [22] and current studies demonstrate that in contrast to other pneumococcal infections, the presence of a capsule does not seem to be an essential component in the pathogenesis of conjunctivitis. Instead of a capsule, the ECC strains carry a variety of novel or modified surface proteins that correlate with the remarkable hyperspecialization of these strains to the conjunctival tissue, and appear likely to play a role in the diseases pathogenesis [22]. Even among non-ECC-related isolates that are commonly encapsulated and do not carry the unique ECC surface features, we found potential evidences for selection against the presence of capsule in the conjunctiva. Six non-ECC isolates were recovered from conjunctivitis in our study, with half of them being non-typeable. On the other hand, given that most (95.2%) of the pneumococcal isolatescausing keratitis were encapsulated, the presence of an anionic polysaccharide capsule that enables the bacterial cell to evade phagocytic clearance, appears to be a key factor in the development of corneal infection. Despite the anatomical proximity of the conjunctiva and cornea, our results point to the existence of relevant host-derived and niche-specific components that governs the microscale biogeography of ocular surface pneumococcal infections, resulting in the selection of significantly distinct populations of *S. pneumoniae* isolatesin these eye compartments.

Among ECC isolates (*n*=10), isolates belonging to ST448 were the most common in our study (*n*=9). This lineage has been implicated in many outbreaks of pneumococcal conjunctivitis in the USA since 1980 [20] and has become established as a persistent lineage that is trophic for, and well adapted to eye tissues [22]. The largest reported outbreak occurred among 698 college students over a 3 month period in 2002 at Dartmouth College. Analysis of the epidemiology of the outbreak showed that the ST448 strain was highly transmissible and quickly spread [20]. Subsequent outbreaks caused by this strain were reported in schools, childcare centres and colleges [3, 38, 39]**.** The ST448 strain seems to have become stably established in the community and is the major cause of non-outbreak-related pneumococcal conjunctivitis in the USA [18, 22], but nevertheless rarely, if ever, infect the cornea.

The widespread use of pneumococcal vaccines has significantly decreased the morbidity and mortality of invasive diseases caused by vaccine serotypes [40]. However, this selective pressure is known to result in increased carriage by replacement non-vaccine serotypes [33]. In our sample representing a post-PCV-13 population, infectious keratitis was caused by a highly diverse group of isolates that are mainly encapsulated (95.2 %), with the majority of them not covered by the PCV-13. This heterogeneous population was formed by isolates grouping within a variety of CCs with no apparent clonal dominance, carried 13 different capsule operon types, and appears to mirror the current post–PCV-13 strain compositions in invasive diseases [41] and carriage [42], where PCV-13 serotypes other than serotype 3 are relatively uncommon. Metcalf *et al.* characterized IPD isolates collected from a multistate surveillance study in the USA during 2018, and found that non-PCV-13 serotypes accounted for more than 70 % of the isolates [43]. In our study only one keratitis isolate carried a capsule operon inferred as a PCV-13 serotype (19A). Interestingly, based on our findings, the recently approved 20-valent PCV could potentially expand vaccine coverage in keratitis and could have helped prevent eight additional cases caused by the serotypes 15B (*n*=2), 11A (*n*=2), 33F (*n*=1), 22F (*n*=2) and 10A (*n*=1). Pneumococcal keratitis can be a serious and sight-threatening infection associated with significant morbidity [4, 44–46]. Although the burden of this infection likely could be reduced by vaccination, our data demonstrate that the current vaccine designs leave gaps that non-vaccine capsule types can exploit.

Given the high diversity of capsular serotypes in the *S. pneumoniae* keratitis population, preventive approaches that rely on the development of updated PCVs targeting the capsule may not be feasible. In addition, the wide range of capsular serotypes that can be expressed by *S. pneumoniae* colonizing humans associated with its natural transformability and frequent capsule switching events, may further complicate the design of PCVs aiming to reduce the burden of pneumococcal keratitis. Additional designs targeting other central virulence factors, for which a more restricted number of structural variants is biologically conceivable, may result in more universal vaccines that could potentially be less impacted by constant recombination events [47]. Since virulence studies have indicated that the pathogenicity of pneumococcal keratitis is mainly caused by the ability of pneumolysin to induce a massive inflammatory response [48], neutralizing this exotoxin could be an effective strategy to prevent and treat pneumococcal keratitis as already demonstrated in animal models [49–51].

Among the antimicrobial classes commonly used for treatment of Gram-positive keratitis, our population was sensitive to the fluoroquinolones and vancomycin, and showed different degrees of resistance to macrolides including erythromycin and azithromycin. Resistance to macrolides in *S. pneumoniae* isolated in the USA is relatively common, with rates ranging from 20–40 % [52]. Over a period of 4 years (2014–2017), we detected an increase in macrolide resistance, with twice as much resistant isolates in 2017 compared to 2014. We found that resistance to macrolides in our ocular *S. pneumoniae* population was most commonly mediated by the ABC-type efllux system MefA/MsrD, which was the dominant macrolide resistance genotype of *S. pneumoniae* in isolates causing conjunctivitis characterized in our previous report (particularly STs 448 and 1186) and also from other infectious sites in the USA [53]. This efflux system confers resistance to 14- and 15-membered (M phenotype), but not to 16-membered macrolides [54]. In agreement with this, our isolates exhibited low-level *in vitro* resistance to erythromycin, azithromycin and clarithromycin, but not to tylosin. Less frequently, macrolide-resistant isolates carried *ermB* gene (*n*=4), encoding ribosomal methylases. *erm*B carriage resulted in high-level resistance to the macrolides tested (MIC>32 µg ml^−1^), and was co-located with the *tet*M gene in a Tn-916-like transposon. The genetic environment of this transposon was similar to what we had previously found among unencapsulated strains causing conjunctivitis [22]. No significant difference was found in the carriage of acquired antibiotic resistance elements between encapsulated and unencapsulated isolates.

In summary, we found that isolates grouped within the ECC are highly adapted and mainly confined to the conjunctival mucosa with ST448 being most frequently identified. Conversely, *S. pneumoniae* keratitis were caused by a highly diverse population of isolates grouping within 15 different clonal complexes. This genotypically heterogeneous population of keratitis isolates, also presents with a high degree of diversity at the capsule serotype level, with most of the serotypes not being targeted by PCV-13. Overall, 95.5 % of our ocular *S. pneumoniae* population was not targeted by existing vaccines. Because of the extensive variation observed, modification of vaccines targeting conventional *S. pneumoniae* virulence traits such as the polysaccharide capsule may not provide coverage to prevent most of the ocular infections seen in our service. These data will be useful in informing the design of next-generation vaccines targeting other virulence factors that are central in the pathogenesis of ocular infections, which could help decrease the economical and health burden of pneumococcal eye infections.
